# West Nile virus in overwintering Culex mosquitoes, New York City, 2000.

**DOI:** 10.3201/eid0704.010426

**Published:** 2001

**Authors:** R S Nasci, H M Savage, D J White, J R Miller, B C Cropp, M S Godsey, A J Kerst, P Bennett, K Gottfried, R S Lanciotti

**Affiliations:** CDC-DVBID, PO Box 2087, Fort Collins, CO 80522, USA. rsn0@cdc.gov

## Abstract

After the 1999 West Nile (WN) encephalitis outbreak in New York, 2,300 overwintering adult mosquitoes were tested for WN virus by cell culture and reverse transcriptase-polymerase chain reaction. WN viral RNA and live virus were found in pools of Culex mosquitoes. Persistence in overwintering Cx. pipiens may be important in the maintenance of WN virus in the northeastern United States.


### West Nile Virus in Overwintering *Culex* Mosquitoes, New York City, 2000

**Roger S. Nasci,* Harry M. Savage,* Dennis J. White,† James R. Miller, ‡ Bruce C. Cropp,* Marvin S. Godsey,* Amy J. Kerst,* Paul Bennett, § Kristy Gottfried,* and Robert S. Lanciotti***

*Centers for Disease Control and Prevention, Fort Collins, Colorado, USA; †New York State Department of Health, Albany, New York, USA; ‡New York City Department of Health, New York, New York, USA; and §New York City Department of Environmental Protection, New York, New York, USA

After the 1999 West Nile (WN) encephalitis outbreak in New York, 2,300 overwintering adult mosquitoes were tested for WN virus by cell culture and reverse transcriptase-polymerase chain reaction. WN viral RNA and live virus were found in pools of *Culex* mosquitoes. Persistence in overwintering *Cx. pipiens* may be important in the maintenance of WN virus in the northeastern United States.

The 1999 outbreak of human encephalitis in New York City (1) due to infection with West Nile (WN) virus (2) represented the first documented introduction of this virus into the Western Hemisphere. After the outbreak, the Centers for Disease Control and Prevention (CDC) and the U.S. Department of Agriculture recommended that surveillance efforts be enhanced in areas from Massachusetts to Texas along the Atlantic and Gulf coasts (3,4). Of primary concern was the lack of information about the ability of WN virus to persist over the winter in the northeastern United States and reinitiate enzootic or epidemic transmission in spring 2000. Evidence of persistent WN virus transmission in Romania for at least 2 years following the 1996 epidemic (5) increased concern that WN virus would persist and become established in the United States. Since the New York outbreak occurred in an area where mosquito biting activity ceases during winter months, survival of virus-infected female mosquitoes was considered the most likely mechanism for the virus to survive through the winter. Vertical transmission of WN virus by mosquitoes (i.e., passage of virus from infected female to her offspring) has been demonstrated in the laboratory (6) and apparently occurs by entry of virus into mosquito eggs during oviposition (7). Vertical transmission of WN virus has been documented only once in a population of mosquitoes outside the laboratory (8). We describe collection of overwintering mosquitoes during January and February 2000 in New York City and detection of WN viral RNA and live WN virus in the specimens.

### The Study

Numerous sites characteristic of harborage for overwintering adult *Culex* mosquitoes in New York City were visited during January 11-13, February 15-16, and February 25, 2000. These sites were concentrated in northern Queens and southern Bronx, where WN virus activity was detected in mosquitoes during 1999 (9). We suspected that the vast sanitary and storm sewer systems in New York City would harbor large populations of overwintering adult mosquitoes. We sampled pipe chases, pump buildings, and dewatering facilities at the Tallman Island sewage treatment facility in Queens and the Hunts Point sewage treatment facility in the Bronx. In addition, we searched for mosquitoes in 15 manholes leading to sanitary and combined sewers, 31 storm sewer catch basins, and 4 large-diameter (1.2 to 2.5 m) storm water outflow pipes in Queens and the Bronx. Other sites included unheated structures associated with utility equipment rooms under the south end of the Whitestone Bridge; pump service buildings and pipe chases associated with municipal swimming pools in Astoria Park, Crotona Park, and Van Cortlandt Park; abandoned buildings at Flushing Airport; the basement of a historical house in Van Cortlandt Park; and historical structures associated with "The Battery" at Fort Totten in Queens.

Adult mosquitoes were located with a flashlight and collected from walls and ceilings of the resting sites by a large, battery-powered backpack aspirator or small hand-held mechanical aspirator. The specimens were held for 24 to 72 h at 21 to 22°C with access to 5% sucrose solution. Dead specimens were removed from the holding cages, frozen as soon as possible after death, and placed in labeled tubes at -70°C. Surviving specimens were frozen, placed in labeled tubes, held at -70°C, and, along with dead specimens, were shipped to the laboratory of CDC's Division of Vector-Borne Infectious Diseases in Fort Collins, CO. The mosquitoes were identified to species if possible, but the condition of certain morphologically similar *Culex* mosquitoes often prevented identification to species level. As a result, many specimens were only identified to genus or species group (e.g., the *Culex* species category may include the morphologically similar species *Cx. pipiens* and *Cx. restuans*). Specimens were grouped into pools of up to 50 mosquitoes by species, date, and location of collection.

A total of 2,383 adult mosquitoes were collected, 2,380 of which were in the genus *Culex;* the pools also included one

Address for correspondence: Roger S. Nasci, CDC-DVBID, P.O. Box 2087, Fort Collins, CO 80522, USA; fax: 970-221-6476; e-mail: rsn0@cdc.gov adult *Cx. territans* and one *Cx. erraticus* (Table). The other specimens were *Anopheles punctipennis* or unidentified *Anopheles* species. Structures associated with the sanitary and storm sewer systems produced very few specimens. This discovery was unexpected because hibernating *Cx. pipiens* in peridomestic habitats use storm sewers, basements, unheated outbuildings, and similar protected sites (10). Approximately 88% of the *Culex* mosquitoes came from structures built into hillsides and the battery structures constructed of heavy granite block or concrete at Fort Totten.

The 2,383 mosquitoes were separated into 91 pools for testing, and every pool was screened for viable virus by a Vero cell plaque assay (11). They were also tested for WN viral RNA by WN virus-specific reverse transcriptase-polymerase chain reaction (RT-PCR) and a TaqMan RT-PCR assay (12). No evidence of live virus was observed in any of the pools in the initial Vero cell plaque assay, nor was WN viral RNA detected with the traditional RT-PCR assay. Three of the pools containing mosquitoes morphologically identified as *Culex* species tested positive by the TaqMan RT-PCR assay, indicating the presence of WN virus RNA. The TaqMan RT-PCR WN virus detection procedure has been shown to be more sensitive than traditional PCR and at least as sensitive as the Vero cell plaque assay (12).

The three WN viral RNA-positive pools were subsequently tested for viable WN virus by various techniques. Supernatants from the mosquito pool homogenates were inoculated into cultures of Vero cells, C6/36 *Aedes albopictus* cells, AP/61 *Ae. pseudoscutellaris* cells, and baby hamster kidney (BHK) cells. Flasks (25 $mm^2$) containing confluent monolayers of cells were inoculated with 0.1 mL of the mosquito pool supernatant and incubated for 1 h at 37°C and 28°C for mammal and mosquito cells, respectively. The appropriate maintenance medium was then added to the flasks. The cells were incubated and observed daily for cytopathic effects (CPE) for 7 days. Cells were harvested on day 7 regardless of CPE, pelleted by centrifugation, resuspended in phosphate-buffered saline (pH 7.2), and used to prepare spot slides (15 µL of cell suspension per spot). The slides were dried, fixed in cold acetone (-20°C for 20 min), and examined with indirect fluorescent antibody (IFA) staining using Broad Group B and WN virus specific antiserum (2,13). Negative cells of each type were included as controls. In addition, adult *Cx. pipiens* mosquitoes were inoculated with 0.34 µL of mosquito pool supernatant and incubated for 7 days at 27°C (14). Ten mosquitoes were inoculated with each of the three pool supernatants. The mosquitoes were then tested for virus by triturating them in BA-1 diluent as described for the Vero cell plaque assay, and injecting supernatants from the triturates into Vero cell cultures. The cells were observed for evidence of cytopathic effect for 7 days then harvested and examined with IFA stain as described. Live WN virus was isolated from the supernatant of one of the three RNA-positive pools inoculated into Vero cell culture. None of the other attempts to isolate virus from these pools were successful. The difficulty in isolating live virus from the RNA-positive pools, despite extensive efforts, may be due to virus death during the collection and shipping process or to a naturally low virus titer in vertically infected, hibernating mosquitoes.

The identity of mosquitoes in two of the WN viral RNA-positive pools was subsequently determined by a species-diagnostic PCR assay that can differentiate between *Cx. pipiens*, *Cx. restuans*, and *Cx. salinarius* in the pool (15). Results indicated that the two pools contained only *Cx. pipiens*. Insufficient material was available from the third RNA-positive pool for species identification by PCR.

Table. Adult mosquitoes collected in overwintering sites, Queens and the Bronx, January and February 2000

| Borough | Site | Species | No. mosquitoes |
|---|---|---|---|
| Queens | Tallman Island Sewage Plant | *Culex pipiens* | 1 |
| | | *Cx. restuans* | 4 |
| | | *Cx.* species | 74 |
| | Fort Totten | *Cx. pipiens*[a] | 1,034 |
| | | *Cx. restuans* | 11 |
| | | *Cx. erraticus* | 1 |
| | | *Cx. territans* | 1 |
| | | *Cx.* species[b] | 1,045 |
| | | *Anopheles punctipennis* | 2 |
| | | *An.* species | 1 |
| | Other sites combined | *Cx. pipiens* | 24 |
| | | *Cx. restuans* | 4 |
| The Bronx | Hunts Point Sewage Plant | *Cx.* species | 33 |
| | Other sites combined | *Cx. pipiens* | 145 |
| | | *Cx.* species | 3 |
| | Total | | 2,383 |

[a]West Nile (WN) viral RNA detected in two pools of specimens initially morphologically identified as *Culex* species and subsequently identified as *Cx. pipiens* by species-specific polymerase chain reaction (PCR). Live WN virus was isolated from one of these pools.
[b]WN viral RNA detected in one pool of specimens morphologically identified as *Cx.* species. Insufficient material was available to permit species identification by PCR.

### Conclusions

Detection of WN viral RNA in three pools and isolation of live WN virus from one pool of overwintering *Cx. pipiens* mosquitoes in New York City indicated that WN virus persisted in vector mosquitoes at least through midwinter, suggesting that the virus would persist until spring and emerge with mosquitoes to reestablish an enzootic transmission cycle in the area. Transovarial transmission of WN virus and preservation of the virus in hibernating mosquitoes are not thought to play an important role in the maintenance of the virus in nature (16,17). However, our observations indicate that approximately 0.04% of the overwintering *Culex* mosquitoes collected at Fort Totten carried viable WN virus, and 0.1% contained WN viral RNA. This finding suggests that WN virus infected, hibernating *Cx. pipiens* were relatively common where virus activity was intense the previous season and likely play an important role in persistence of the virus in an area. This infection rate is similar to rates observed for another flavivirus, St. Louis encephalitis virus, in overwintering *Cx. pipiens* collected in Maryland, where 0.3% were infected (1 isolate from 312 tested), and Pennsylvania, where 0.2% were infected (1 isolate from 406 tested) (18).

What is unclear is the mechanism that produced these infected overwintering mosquitoes. Transovarial transmission of the virus from an infected female to her offspring, which then enter diapause (hibernation physiology and behavior) as adults and survive the winter without taking a blood meal, is supported by evidence from the field and laboratory (6,8). Alternatively, *Cx. pipiens* infected by feeding on a viremic vertebrate host may have survived the winter. Though blood-fed adult *Cx. pipiens* survive winter conditions (19), they are not considered an efficient mechanism for virus persistence (10). Regardless of the underlying mechanism, WN virus persistence in *Cx. pipiens* clearly contributes to the maintenance of WN virus through the winter season. Future research should address the mechanisms of WN virus maintenance and potential involvement of other mosquito species that may be important vectors in other regions of North America.

### Acknowledgments

We thank Frank Vazquez and other staff of the New York City Department of Environmental Protection, the New York City Mayor's Office of Emergency Management, and the New York City Fire Department for access to collection sites and Joanne Oliver, Robert Means, Neeta Pardanani, and Sara Bumberger for assistance in collecting specimens.

Dr. Nasci is research entomologist at the Division of Vector-Borne Infectious Diseases, Centers for Disease Control and Prevention, Fort Collins, Colorado. His research interests include the ecology and control of mosquito-transmitted zoonoses.

### References

1. Centers for Disease Control and Prevention. Update: West Nile Virus encephalitis—New York 1999. MMWR Morb Mortal Wkly Rep 1999;48:944-6,955.
2. Lanciotti RS, Roehrig JT, Deubel V, Smith J, Parker M, Steele K, et al. Origin of the West Nile virus responsible for an outbreak of encephalitis in the northeastern United States. Science 1999;286:2333-7.
3. Centers for Disease Control and Prevention. Guidelines for surveillance, prevention, and control of West Nile virus infection—United States. MMWR Morb Mortal Wkly Rep 2000;49:25-8.
4. Gubler DJ, Campbell GL, Nasci R, Komar N, Petersen L, Roehrig JT. West Nile Virus in the United States: Guidelines for detection, prevention, and control. Viral Immunol 2000;13:469-75.
5. Cernescu C, Nedelcu NI, Tardei G, Ruta S, Tsai TF. Continued transmission of West Nile virus to humans in southeastern Romania, 1997-98. J Infect Dis 2000;181:710-2.
6. Baqar S, Hayes CG, Murphy JR, Watts DM. Vertical transmission of West Nile virus by *Culex* and *Aedes* species mosquitoes. Am J Trop Med Hyg 1993;48:757-62.
7. Rosen L. Further observations on the mechanism of vertical transmission by *Aedes* mosquitoes. Am J Trop Med Hyg 1988;39:123-6.
8. Miller BR, Nasci RS, Godsey MS, Savage HM, Lutwama JJ, et al. First field evidence for natural vertical transmission of West Nile virus in *Culex univitattus* complex mosquitoes from Rift Valley Province, Kenya. Am J Trop Med Hyg 2000;62:240-6.
9. Nasci RS, White DJ, Stirling H, Oliver JA, Daniels TJ, Falco RC, et al. West Nile virus isolates from mosquitoes in New York and New Jersey, 1999. Emerg Infect Dis 2001;7:626-30.
10. Mitchell CJ, Francy DB, Monath TP. Arthropod Vectors. In: Monath TP, ed. St. Louis Encephalitis. Washington DC: American Public Health Assn; 1980. p. 313-79.
11. Beaty BJ, Calisher CH, Shope RS. Arboviruses. Schmidt NJ, Emmons RW, eds. Diagnostic procedures for viral, rickettsial and chlamydial infections. Washington DC: American Public Health Assn; 1989. p. 797-856.
12. Lanciotti RS, Kerst AJ, Nasci RS, Godsey MS, et al. Rapid detection of West Nile virus from human clinical specimens, field collected mosquitoes, and avian samples by a TaqMan RT-PCR assay. J. Clinical Microbiol 2000;38:4066-71.
13. Lorono-Pina MA, Cropp CB, Ferfan JA, Vorndam VA, Rodrigiez-Angulo EM, et al. Common occurrence of concurrent infections by multiple dengue virus serotypes. Am J Trop Med Hyg 1999;61:725-30.
14. Rosen L, Gubler, DJ. The use of mosquitoes to detect and propagate dengue viruses. Am J Trop Med Hyg 1974;23:1153-60.
15. Crabtree MB, Savage HM, Miller BR. Development of a species-diagnostic polymerase chain reaction assay for the identification of *Culex* vectors of St. Louis encephalitis virus based on interspecies sequence variation in ribosomal DNA spacers. Am J Trop Med Hyg 1995;53:105-9.
16. Taylor FM, Work TH, Hurlbut HS, Rizk F. A study of the ecology of West Nile virus in Egypt. Am J Trop Med Hyg 1956;5:579-620.
17. Malik Peris JSM, Amerasinghe FP. West Nile Fever. In: Beran GW, Steele JH, editors. Handbook of Zoonoses, Second Edition, Section B: Viral. Ann Arbor, MI: CRC Press; 1994. p. 139-48.
18. Bailey CL, Eldridge BF, Hayes DE, Watts DM, Tammariello RF, Dalrymple JM. Isolation of St. Louis encephalitis virus from overwintering *Culex pipiens* mosquitoes. Science 1978;199:1346-9.
19. Bailey CL, Faran ME, Gargan TP, Hayes DE. Winter survival of Blood-fed and nonblood-fed *Culex pipiens* L. Am J Trop Med Hyg 1982;31:1054-61.
